# Social, demographic and healthcare factors associated with stage at diagnosis of cervical cancer: cross-sectional study in a tertiary hospital in Northern Uganda

**DOI:** 10.1136/bmjopen-2015-007690

**Published:** 2016-01-21

**Authors:** Amos Deogratius Mwaka, Christopher Orach Garimoi, Edward Maloba Were, Martin Roland, Henry Wabinga, Georgios Lyratzopoulos

**Affiliations:** 1 Department of Medicine, School of Medicine, College of Health Sciences, Makerere University, Kampala, Uganda; 2 Department of Community Health, School of Public Health, College of Health Sciences, Makerere University, Kampala, Uganda; 3 Department of Management Science for Health, Uganda; 4 Department of Health Services Research, Institute of Public Health, University of Cambridge, Cambridge, UK; 5 Department of Pathology, Kampala Cancer Registry, College of Health Sciences, Makerere University, Kampala, Uganda

**Keywords:** Cervical cancer, predictors of late stage, pathways to diagnosis and treatment

## Abstract

**Objective:**

To examine patient and primary healthcare factors and stage at diagnosis in women with cervical cancer in Northern Uganda with the intention to identify factors that are associated with advanced stages in order to inform policies to improve survival from cervical cancer in low income and middle income countries.

**Design:**

Cross-sectional hospital-based study.

**Setting:**

Tertiary, not-for-profit private hospital in postconflict region.

**Participants:**

Consecutive tissue-diagnosed symptomatic patients with cervical attending care. Of 166 patients, 149 were enrolled and analysed.

**Primary outcome:**

Cervical cancer stage at diagnosis.

**Results:**

Most women were diagnosed at stages III (45%) or IV (21%). After controlling for age, marital status, educational attainment and number of biological children, there was evidence for association between advanced stage at diagnosis and pre-referral diagnosis of cancer by primary healthcare professionals (adjusted OR (AOR)=13.04:95% CI 3.59 to 47.3), and financial difficulties precluding prompt help seeking (AOR=5.5:95% CI 1.58 to 20.64). After adjusting for age, marital status and educational attainment, women with 5–9 biological children (AOR=0.27:95% CI 0.08 to 0.96) were less likely to be diagnosed with advanced stage (defined as stages III/IV) cancer. In this pilot study, there was no statistical evidence for associations between stage at diagnosis, and factors such as age at diagnosis and marital status.

**Conclusions:**

This study is a first attempt to understand the descriptive epidemiology of cervical cancer in rural Ugandan settings. Understanding individual patient factors, patients’ behavioural characteristics and healthcare factors associated with advanced stage at diagnosis is essential for targeted effective public health interventions to promote prompt health seeking, diagnosis at early stage and improved survival from cervical cancer.

**Strengths and limitations of this study**

- This is a pioneering study in a low income country to apply the theoretical framework—the Model of Pathways to Treatment—to evaluate factors that may influence symptom appraisal and help-seeking intervals for symptomatic patients with cervical cancer.
- Participants were prospectively recruited thereby minimising methodological concerns associated with retrospective studies.
- Diagnosis of cervical cancer was confirmed by tissue histology and following examinations under anaesthesia, thus obviating possibilities of diagnostic misclassifications.
- Potential recall or social desirability biases are inherent in patient interview studies. Most patients presented long after onset of symptoms and could have had difficulty in recall of some events.
- This was a hospital-based study involving a selected population of women who had reached the hospital. The characteristics of women who may have cervical cancer but who have not reached the study hospital remain unknown.

## Background

In Uganda and most low income and middle income countries (LMICs), there is limited evidence about the distribution of stage at diagnosis of cervical cancer, and about factors that contribute to advanced stage at diagnosis. In Nepal, up to 81% of patients with cervical cancer are diagnosed with advanced stage cancer. Women who were more likely to be diagnosed with advanced stage cancer included those who did not disclose their symptoms to significant others promptly (adjusted OR (AOR)=4.27) and those who disclosed symptoms to relations other than their husbands (AOR=12.70).^1^

A modifiable predictor of treatment outcome is time to diagnosis of symptomatic cervical cancer. Among symptomatically detected women with cervical cancer in Sweden, those with shorter symptom durations had 14% higher chances of cure compared to those with longer symptom duration.^2^ However, in most LMICs, the diagnostic journeys of most women with cervical cancer symptoms are dominated by long patient and primary care intervals. Of 110 symptomatic patients with cervical cancer in Nepal, the median total time to diagnosis (diagnostic interval) was 157 days while the median patient and healthcare provider intervals were 68.5 days and 40 days, respectively. Fifty-seven per cent of the patients had experienced longer patient intervals of >2 months.^3^ Diagnosing cervical cancer at an early stage requires that women recognise and appraise the importance of possible cervical cancer symptoms early and seek care promptly.^4^ Nonetheless, even when women seek care for cervical cancer symptoms, diagnosis may be delayed because primary healthcare professionals face challenges in promptly recognising symptoms and referring patients with possible cervical cancer.^5^ ^6^ In Uganda, data from the Kampala Cancer Registry (which serves a population in close proximity to specialised cancer treatment centres) suggest that women in central Uganda present with advanced stage cervical cancer and have poor prognosis.^7^ ^8^ Previous studies, however, have not been able to examine factors that are likely to influence the promptness of diagnosis; such factors might vary within and between countries and regions. Understanding of context-specific factors including patient-related and primary healthcare-related factors that lead to delay in a particular country can guide development of targeted interventions and policies to increase prompt appraisal of cervical cancer symptoms, and enable timely help seeking.

In spite of cervical cancer being a very common cancer, responsible for 2275 deaths every year (27.2 deaths per 100 000 women), women with cervical cancer in Uganda are an understudied population.^9^ Against this background, we set out to examine patient and primary healthcare factors, and stage at diagnosis in women with cervical cancer in Northern Uganda, with the intention of establishing the feasibility of future larger studies aimed at identifying factors associated with advanced stages in broader populations.

## Methods

### Theoretical framework

**Figure 1:**
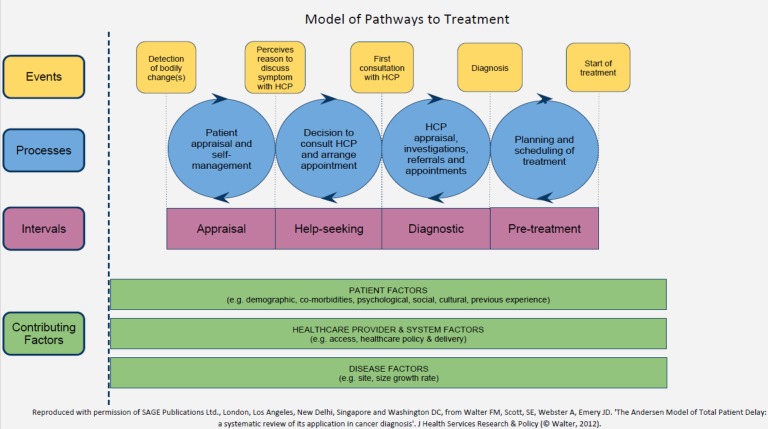
Model of Pathways to Treatment. HCP, healthcare professional.

The data collection and analysis in this study were underpinned by the Model of Pathways to Treatment (MPT).^10^ ^11^ In this model (figure 1), the cancer journey from symptom recognition through help seeking, diagnosis and treatment, is viewed as an iterative process composed of events and processes with distinguishable intervals. These events and associated intervals are influenced by factors such as patient demographics, healthcare access and disease factors, including rate of progressions and histological subtypes. In using the MPT, researchers and policymakers can gain insight into actual points along the journey where delay may occur and hence provide opportunities for design of targeted interventions.^11^

The operational definitions for different measures and markers of promptness of diagnosis used in this study are presented in table 1. These definitions were informed by the MPT model, and an international consensus statement on the measurement of different diagnostic intervals^12^ and evidence indicating that the number of pre-referral consultations is a valid marker of the primary care interval.^13^

**Table 1:** Operational definitions

Term/concept	Definition	Author
Date of first symptom	The date or estimated time in the week, month or year when the patient first felt a bodily change requiring discussion with a healthcare professional and/or with another person with the intention of gaining understanding of the symptoms and/or how to deal with them	Weller D, *et al*^12^
Patient interval	The time period from detection of abnormal bodily sensations to time of visiting first healthcare professional to discuss the symptoms including the period from lower level units to the study hospital for diagnosis of cancer	Weller D, *et al*^12^
Date of first presentation	The time point in the week, month or year including a particular date when the patient first visited a healthcare professional in a private or public health facility to discuss the symptoms that she had and that have since been attributed to cervical cancer at the study hospital	Weller D, *et al*^12^
Date of diagnosis	Date when examination under anaesthesia for clinical diagnosis and staging was performed.	Weller D, *et al*^12^
Pre-referral consultations	Any visits to a healthcare professional in an established healthcare setting including lower level healthcare facilities and private clinics before presentation to and diagnosis at the study hospital	Lyratzopoulos G, *et al*^13^
Pre-referral suspicion or diagnosis of cancer	Any reports by participants referring to being told of a cancer diagnosis by primary healthcare professionals before referral to study hospital	

### Design and setting

We carried out a cross-sectional survey of patients with cervical cancer attending St Mary's Hospital Lacor—a tertiary 400-bed not-for-profit hospital in northern Uganda. This was a pragmatic feasibility study to increase knowledge and help pave the way for the conduct of larger multisite studies.

### Participants and recruitment

We consecutively recruited all women with cervical cancer diagnosed and treated in the gynaecology department over the study period of 1½ years. In all women, the diagnosis was confirmed with tissue histology and stage was assigned based on the findings of examination under anaesthesia (EUA), carried out by a gynaecologist. Staging was performed according to the International Federation of Gynaecology and Obstetrics (FIGO) staging criteria.^14^ Sampling was confined to women diagnosed within a maximum period of 6 months from the date of recruitment.

Screening-detected women were a priori excluded. Patients who could not be interviewed because of language problems were also excluded. Altogether, 17 of 166 women seen in the hospital during the study period were excluded; five were diagnosed more than 6 months before study recruitment, two declined to participate because they wanted to go immediately for radiotherapy in the capital city, Kampala, four unexpectedly left hospital soon after EUAs and five were too sick to be interviewed. An 18-year-old patient with EUA report showing a fungating mucinous cervical mass was excluded because histopathology revealed diagnosis of sarcoma botryoides. The sample therefore included 149 women.

### Data collection

Data were collected from the patients, using structured interviews based on a questionnaire administered by a research assistant. We designed the questionnaire based on contributing factors, events and intervals in the Model of Pathways to Treatment, findings from studies on cervical cancer in sub-Saharan Africa and our own clinical experiences.^11^ ^15^ The questionnaire had three sections: sociodemographic characteristics; knowledge and beliefs about cervical cancer including awareness of risk factors, symptoms and beliefs about treatment and cure; and information about care processes including symptoms appraisal, duration and help-seeking behaviour. The questionnaire also included open-ended questions about symptoms, the distance from home to nearest health unit and to the study hospital, and the number of times health units were visited before coming to the study hospital. Responses from the open-ended questions were reviewed and aggregated before further analysis. Symptoms that were reported by fewer than five participants were categorised as ‘other’ and excluded from the main analyses.

The study tool was double translated by two independent translators fluent in English and Luo/Acholi, the main local language spoken in the study area. A third translator reviewed both Luo versions and harmonised them to form the final tool used in data collection.

A female registered nurse with additional and midwifery qualification was trained as research assistant for this study. The research assistant was trained for 2 days on basic facts on cervical cancer and the study procedures including criteria for inclusion, consent procedures and data collection. The research assistant introduced the study to prospective participants after the patients were informed of their cancer diagnoses by the attending physicians as part of their standard care. After at least 5 days from the disclosure of cancer diagnoses, the research assistant administered the questionnaire, mostly in the local language. The research assistant read out the questions and coded responses to the participants, and ticked and/or recorded responses accordingly. However, for the questions concerning risk factors and perceived causes, the research assistant did not prompt recall by reading out the options but ticked all coded responses as the participants mentioned them. She prompted participants to mention as many risk factors/perceived causes as possible by asking, “what else do you think may also cause this cancer?” This was performed to avoid suggesting risk factors that participants would not have thought about. Administration of questionnaires lasted about 45–60 min. The research assistant abstracted clinical data such as cancer stage and histology from the patients’ case notes and histopathology laboratory record. Recruitment and data collection were conducted from September 2012 to April 2014.

### Data analysis

Data entry was performed by two independent clerks using Epidata V.3.1 software and analysis carried out using STATA I/C V.12.0. The outcome measure was cancer stage at diagnosis, dichotomised as early (I/II) or advanced stage (III/IV). Independent variables/covariates included sociodemographic characteristics, time to diagnosis, estimated road distance from study hospital, initial symptom attributions, pre-referral diagnoses by primary healthcare professionals, number of pre-referral visits, health-seeking intervals and reported reasons for non-prompt health seeking. Bivariate and multivariable logistic regressions were used to determine associations. For multivariable regression analysis, variables were included based on biological plausibility rather than a predetermined p value in bivariate analyses. Odd ratios and accompanying 95% CIs are reported.

### Ethical clearance

Participants were informed of study objectives, consent procedures and potential benefits and harms, and their right to decline participation and/or withdraw at any time without fear of retribution or compromise to their cancer management plans. All participants provided informed individual consents with a signature or thumb print.

## Results

### Characteristics of participants

One hundred forty-nine women with cervical cancer were included in the analysis. The mean age (±SD) was 48±13 years. Fifty-seven per cent of participants were married; 45% reported no formal education and 89% were not formally employed. Seventy-two per cent of participants had five children or more while 39% of participants lived more than 100 km from the study hospital (median distance=80 km, range; 2–375 km). Most participants had stage III (45%) or IV (21%) disease at diagnosis. Squamous cell carcinoma (75%) was the predominant histological subtype of cancer identified (table 2).

**Table 2:** Demographic characteristics of patients and disease characteristics

Characteristics	Number (N=149)	Percentage
Age group (years)		
18–29	7	4.7
30–44	52	34.6
45–59	63	42.7
≥60	25	16.7
Missing	2	1.3
Mean age (±SD) years	48.4±12.6	
Median age	49.0 (23–80)	
Marital status		
Married	84	56.4
Divorced	21	14.1
Widowed	44	29.5
Mean age at marriage (years)	17.7±2.3	
Median age 18 (13–27) years		
Education attainment		
No formal education	67	45.0
Primary education	72	48.3
Secondary education	7	4.7
Tertiary education	2	1.3
Missing	1	0.7
Occupation		
Housewife/peasant	132	88.6
Petty trader	10	6.7
Formally employed	4	2.7
Missing	3	2.0
Number of biological children		
No child	2	1.3
1–4	28	18.7
5–10	108	72.0
11–15	10	6.7
Missing	1	0.7
Stage of cancer at diagnosis (FIGO)		
Stage I	17	11.4
Stage II	29	19.5
Stage III	67	45.0
Stage IV	31	20.8
Missing	5	3.3
Histological subtypes		
Squamous cell carcinoma	111	74.5
Adenocarcinoma	12	8.1
Anaplastic type	1	0.7
Missing	25	16.7
Estimated distance from home to study hospital (Kilometres)		
Less than 40	41	27.5
40–80	35	23.5
81–100	13	8.7
101–375	58	38.9
Missing	2	1.3
Median (range)	80 (2–375)	

FIGO, International Federation of Gynaecology and Obstetrics.

### Patient-related factors and cancer stage at diagnosis

#### Participants’ sociodemographics

In bivariate analyses, participants with secondary and tertiary education were less likely to be diagnosed with advanced stage cancer compared to those who had not attained formal education (crude OR=0.16 (95% CI 0.03 to 0.87). After adjusting for age, marital status and educational attainment, the odds of advanced stage cancer among patients with 5–9 children was 0.27 (95% CI 0.08 to 0.96) times the odds of advanced cancer among women with less than four children (table 3).

**Table 3:** Adjusted OR for patients’ sociodemographic characteristics and stage at diagnosis

		Cancer stage at diagnosis			
		Early stage	Advance stage		
Patient demographic characteristics	Population responding	Number (%)	Number (%)	Crude OR (COR) (95% CI)	Adjusted OR (AOR) (95%CI)*
Age group (years)					
<30	6 (4.2)	3 (6.5)	3 (3.1)	1.00	1.00
30–59	111 (78.2)	40 (87.0)	71 (74.0)	1.78 (0.34 to 9.21)	2.62 (0.33 to 21.1)
≥60	25 (17.6)	3 (6.5)	22 (22.9)	7.33 (0.99 to 54.4)	9.82 (0.81 to 118.9)
Marital status					
Married	81 (56.3)	31 (67.4)	50 (51.0)	1.00	1.00
Divorced	19 (13.2)	4 (8.7)	15 (15.3)	2.32 (0.71 to 7.65)	1.81 (0.49 to 6.72)
Widowed	44 (30.5)	11 (23.9)	33 (33.7)	1.86 (0.82 to 4.21)	1.26 (0.51 to 3.12)
Education attainment					
No formal education	67 (48.2)	22 (47.8)	45 (46.4)	1.00	1.00
Primary education	68 (47.6)	18 (39.1)	50 (51.6)	1.36 (0.65 to 2.85)	1.44 (0.62 to 3.34)
Secondary and/or tertiary education	8 (5.6)	6 (13.0)	2 (2.0)	**0.16 (0.03** to **0.87)**	0.18 (0.03 to 1.22)
Number of biological children					
0–4	28 (19.6)	5 (11.1)	23 (23.5)	1.00	1.00
5–9	89 (62.2)	34 (75.6)	55 (56.1)	0.35 (0.12 to 1.01)	**0.27 (0.08** to **0.96)**
10–15	26 (18.2)	6 (13.3)	20 (20.4)	0.72 (0.19 to 2.74)	0.45 (0.1 to 2.09)

Bold typeface indicates statistically significant values.

*Adjusted for age, marital status, education attainment and number of biological children.

#### Initial symptom attribution

Most participants (90.3%) did not attribute their initial symptoms to cervical cancer (table 4). After controlling for age, marital status, educational attainment and number of biological children, the OR of advanced stage cervical cancer among patients who perceived their symptoms as due to a serious illness or cancer was 0.43 times (95% CI 0.20 to 0.96) the OR of those who perceived their symptoms as not due to a serious illness/cancer (table 4).

**Table 4:** Primary care factors and stage at diagnosis

		Cancer stage at diagnosis			
		Early stage	Advanced stage		
Primary care factors	Population responding	Number (%)	Number (%)	Crude OR (COR) (95% CI)	Adjusted OR* (95% CI)
*Symptoms were initially attributed by the patient to*					
Sexually transmitted diseases					
No	121 (84.0)	41 (89.1)	80 (81.6)	1.00	1.00
Yes	23 (16.0)	5 (10.9)	18 (18.4)	1.85 (0.64 to 5.32)	3.24 (0.93 to 11.32)
Cancer					
No	130 (90.3)	39 (84.8)	91 (92.9)	1.00	1.00
Yes	14 (9.7)	7 (15.2)	7 (7.1)	0.43 (0.14 to 1.30)	0.30 (0.08 to 1.16)
*Pre-referral diagnoses by primary healthcare professional*					
Non-cancer related	21 (15.4)	16 (37.2)	5 (5.4)	1.00	1.00
Cancer diagnosis	75 (55.2)	16 (37.2)	59 (63.4)	**11.8 (3.75** to **37.12)**	**13.04 (3.59** to **47.30)**
Not told diagnosis	40 (29.4)	11 (25.6)	29 (31.2)	**8.44 (2.10** to **28.6)**	**8.35 (2.13** to **32.79)**
*Number of pre-referral visits at primary healthcare facilities*					
Once	54 (48.2)	17 (43.6)	37 (50.7)	1.00	1.00
Twice	29 (25.9)	13 (33.3)	16 (21.9)	0.57 (0.22 to 1.43)	0.68 (0.24 to 1.94)
Three to five or more	29 (25.9)	9 (23.1)	20 (27.4)	1.02 (0.39 to 2.70)	0.87 (0.28 to 2.65)
*Health-seeking interval (months)*					
<3	59 (45.4)	23 (53.5)	36 (41.4)	1.00	1.00
3–6	51 (39.2)	15 (34.9)	36 (41.4)	1.53 (0.69 to 3.41)	1.55 (0.62 to 3.86)
7–24	20 (15.4)	5 (11.6)	15 (17.2)	1.92 (0.61 to 5.99)	1.93 (0.52 to 7.23)
*Reasons for lack of promptness in seeking care*					
Lack of money					
No	108 (75.0)	43 (93.5)	65 (66.3)	1.00	1.00
Yes	36 (25.0)	3 (6.5)	33 (33.7)	**7.28 (2.10** to **25.22)**	**5.70 (1.58** to **20.64)**
Still using other treatments					
No	84 (58.3)	23 (50.0)	61 (62.2)	1.00	1.00
Yes	60 (41.7)	23 (50.0)	37 (37.8)	0.61 (0.30 to 1.23)	0.66 (0.30 to 1.43)
Perceived illness as not serious or cancer					
No	86 (59.7)	20 (43.5)	66 (67.4)	1.00	1.00
Yes	58 (40.3)	26 (56.5)	32 (32.6)	**0.37 (0.18** to **0.77)**	**0.43 (0.20** to **0.96)**

Bold typeface indicates statistically significant values.

*OR adjusted for patients’ sociodemographic characteristics in table 3.

#### Socioeconomic factors

After adjusting for the patients’ sociodemographics, patients who reported lack of money as reason for non-prompt health seeking were more likely to be diagnosed at advanced stage cervical cancer while those who perceived their symptoms as serious or due to cancer were less likely to be diagnosed at advanced stage cancer. The odds of advanced stage cancer among patients who self-reported financial difficulty are 5.7 times (95% CI 1.58 to 20.64) the odds of advanced cancer among the patients who did not report financial difficulty as a reason for non-prompt health seeking (table 4).

### Health system factors and cancer stage at diagnosis

About one in three patients with cervical cancer resided >100 km from the study hospital (table 2). About a quarter of the participants attended care 3–5 times before referral (table 4). After controlling for patients’ demographics, the odds of advanced stage cervical cancer among patients who were assigned pre-referral diagnoses of cancer are 13.04 times (95% CI 3.59 to 47.30) the odds among those assigned non-cancer-related diagnoses (table 4).

### Total time to diagnosis at study hospital (appraisal and help-seeking intervals)

More than half (55%) of the participants presented at the study hospital after 3 months or more from reported date of onset of symptoms. Of these, 71.8% (51/71) had advanced stage cervical cancer. Although not statistically significant, participants who took a longer time to presentation tended to be diagnosed at an advanced stage (table 4).

## Discussion

The study provides early insights about the range of psychosocial and healthcare factors that are likely to be associated with prolonged intervals and advanced stage at diagnosis of cervical cancer in Uganda, and should motivate the conduct of multisite studies to establish more independent predictors of advanced stage cervical cancer at diagnoses in LMICs. The findings suggest that several patient characteristics (including age, educational attainment, marital status and number of biological children) and primary healthcare factors may influence diagnostic intervals, and the stage of cervical cancer at diagnosis. More than half of patients with cervical cancer attending care at St. Mary's hospital, Lacor during the study period were diagnosed at an advanced stage.

The odds of advanced stage cancer were higher among older women. Similarly, in Sudan, older age was found to be an independent predictor of advanced stage cervical cancer.^16^ In other LMICs, older women were also found to have long help-seeking intervals for their symptoms and were more likely to be diagnosed with advanced stage cervical cancer.^3^ ^16–18^ Older women are likely to be postmenopausal and may be less keen to seek care promptly for gynaecological symptoms. We also found that women with secondary and tertiary education were less likely to be diagnosed with advanced stage cancer. Similarly, higher level of education was found to be associated with earlier stage at diagnosis in Nepal.^1^ There is evidence that the association between low education level (or other markers of low socioeconomic status) and advanced stage cervical cancer seem to be mediated by early onset of sexual activity among women with low education.^19^

The odds of advanced stage cervical cancer among widowed and divorced women were 1.8–2.3 times the odds among married women. Similar findings were reported in studies conducted in North Africa and India.^18^ ^20^ In the North African study, unmarried women were found to be five times as likely to be diagnosed at an advanced stage compared to married women.^20^ Unmarried women in the USA were also more likely to be diagnosed with advanced stage cervical cancer.^21^ Perhaps married women enjoy emotional and financial support from their spouses and therefore tend to seek help promptly. Evidence indicating a supportive role of husbands is alluded to by findings from Nepal, where women who discussed their symptoms with friends were more likely to be diagnosed in advanced stage compared to those who discussed symptoms with their husbands.^1^ Married women are perhaps less likely to ignore vaginal bleeding and pain because of associated discomfort during sexual intercourse. If married women have a higher frequency of intercourse, this may also lead to a higher symptom burden if the disease is present (eg, painful intercourse, post-coital bleeding), therefore prompting earlier presentation.

Patients’ attribution of their symptoms and perceptions of their likely causes may influence time to help seeking and diagnosis of cancer. We found that patients who initially attributed their symptoms to sexually transmitted diseases (STDs) and/or who never perceived their symptoms as due to a serious illness or cancer, were more likely to be diagnosed with advanced stage cervical cancer compared to those who did not attribute their symptoms to STDs and those who perceived their symptoms as due to a serious illness or cancer. For instance, when adjusted for effect of age, marital status, educational attainment and number of biological children, women who perceived their symptoms to be due to a serious disease or cancer were statistically significantly less likely to be diagnosed at an advanced stage (0.43: 95% CI 0.20 to 0.96) compared to those who did not perceive their symptoms as due to a serious illness or cancer. In a recent systematic review, it was shown that non-recognition of symptom seriousness was related with advanced stage cancer.^22^ Similarly, patients with oral cancers did not initially take their symptoms seriously and attributed such symptoms to common oral conditions for which they responded by self-medication.^23^

Apart from the sociodemographic characteristics of women, advanced stage at diagnosis may relate to other factors that prolong the help-seeking intervals, for example, long distances from diagnostic facilities, lack of money for transport and medical bills, and/or non-recognition of cancer symptoms by primary healthcare professionals. Non-prompt help seeking because of self-reported lack of money was associated with advanced stage at diagnosis. On adjusting for age, marital status, educational attainment and number of biological children, women who reported lack of money as reason for non-prompt help seeking were 5.7 times more likely to be diagnosed with advanced stage cancer compared to those who did not report lack of money as reasons for non-prompt help seeking. Similarly, financial constraint was reported as a main reason for not promptly seeking help in India even among patients who suspected cancer.^24^

Delayed recognition and/or referral by primary healthcare clinicians can nonetheless lead to advanced cancer stage at diagnosis in the referral facilities. In this study, while about half of the patients with cancer received pre-referral diagnoses of cancer at primary healthcare facilities, their primary healthcare professionals did not recognise symptoms and/or suspect cancer in the patients with early stage cancers and referred symptomatic patients with other diagnoses not related to cancer. On adjusting for age, marital status, educational attainment and number of biological children, the odds of advanced cervical cancer among patients assigned pre-referral diagnoses of cancer were 13 times the odds of advanced cancer among women who were assigned other pre-referral diagnoses not related to cancer. In Nepal, healthcare professionals in lower level units made other diagnoses in 90% of the initial pre-referral consultations by patients with cervical cancer symptoms and delayed referral to cancer diagnostic centres.^1^ In a qualitative study in South Africa, primary healthcare professionals were also blamed for prolonged diagnostic intervals in symptomatic women.^6^ Lack of specificity of cervical cancer symptoms and inadequate facilities to aid diagnosis of cervical cancer by healthcare professionals may account for the misinterpretations of cervical cancer symptoms and subsequent diagnosis of non-cervical cancer conditions.^25^

### Strengths and limitations

Strengths to this study include the use of a theoretical model (figure 1) to evaluate factors that may influence symptoms appraisal and help-seeking intervals. Furthermore, participants were prospectively recruited, minimising methodological concerns associated with retrospective studies. Finally, diagnosis of cervical cancer was confirmed by tissue histology and examination following EUAs and this obviates possibilities of diagnostic misclassifications.

The findings are subject to potential recall or social desirability biases inherent in patient interview studies. Most patients presented at the study hospital long after onset of symptoms and could have had difficulty in recall of some events. We had no independent way to ascertain dates of first help seeking in primary care units since records of the lower level health units were not accessible to us for independent verification. However, even when primary care records of patients subsequently diagnosed with cancer were used, there was still a potential for inaccurate measurement of patient interval in particular and, ideally, both primary care and patient-reported data needed to be studied (which was impossible in our setting).^26^ We facilitated recall accuracy by use of calendar landmark techniques, and allowed time between disclosure of cancer diagnosis and questionnaire administration.^27^ In addition, other diseases that present with symptoms similar to what have eventually been diagnosed as cervical cancer could also affect determining the exact time when the actual symptoms of cervical cancer could have started. Measurement of time of onset of symptoms and time intervals during help seeking is known to be challenging.^25^ Second, this was a hospital-based study involving a selected population of women who had reached the hospital. The characteristics of women who may have cervical cancer but who have not reached the study hospital remain unknown. Generalisation of these findings needs to take these issues into consideration.

### Implications of findings

This study provides an initial experience with the conduct of such studies, proving both their feasibility and the need for larger and multisite data collection. Acknowledging power limitations, the findings suggest that advanced stage at diagnosis and long help-seeking intervals could be owing to patients’ misattributions of their symptoms, primary healthcare providers treating cervical cancer symptoms for different common conditions, and a lack of prompt healthcare seeking mainly because of lack of money for transport and medical bills. These findings may have far-reaching implications for clinical care, public health and policy. First, interventions to increase symptoms’ recognition need to target women as well as clinicians, and may take the form of public awareness campaigns and continuous professional development (CPD) for healthcare professionals, respectively. Women who are older, with no or low levels of formal education and widowed/divorced may constitute a special group for interventions to promote prompt help seeking and diagnosis of cervical cancer. Second, to reduce the proportion of advanced stage cervical cancer at diagnoses, policymakers in LMICs ought to prioritise cervical cancer control programmes that include establishment of population-based cervical screening and prompt treatment of preinvasive and early invasive cervical lesions. However, in the meantime, policies on cervical cancer, early detection of preinvasive and early invasive lesions through scheduled CPDs to healthcare professionals, and public awareness campaigns on cervical cancer for the public can be adopted, as they have been shown to be feasible and affordable and can lead to increased survival for those with cervical cancer.^28^

Future studies in Uganda and other LMICs seeking to detect and explain independent predictors of advanced stage cervical cancer may need to include interviews of primary healthcare providers in lower level health facilities in order to provide corroborating evidence and establish reasons for long patient intervals in patients with symptoms of cancer.
